# The FAST VIP (First Aid for Severe Trauma “Virtual” in-Person) Educational Study

**DOI:** 10.5811/westjem.2021.2.50033

**Published:** 2021-06-29

**Authors:** Craig A. Goolsby, Keke Schuler, Raphaelle Rodzik, Nathan Charlton, Vidya Lala, Kevin Anderson, Jeffrey L. Pellegrino

**Affiliations:** * Uniformed Services University of the Health Sciences, Department of Military & Emergency Medicine, Bethesda, Maryland; † Uniformed Services University of the Health Science, National Center for Disaster Medicine and Public Health Medicine, Bethesda, Maryland; ‡ The Henry M. Jackson Foundation for the Advancement of Military Medicine, Inc., Bethesda, Maryland; § University of Virginia School of Medicine, Department of Emergency Medicine, Charlottesville, Virginia; ¶ Uniformed Services University of the Health Sciences, School of Medicine, Bethesda, Maryland; || University of Akron, Department of Disaster Sciences and Emergency Services, Akron, Ohio

## Abstract

**Introduction:**

Trauma is the leading cause of death for young Americans. Increased school violence, combined with an emphasis on early hemorrhage control, has boosted demand to treat injuries in schools. Meanwhile, coronavirus disease 2019 (COVID-19) has made educating the public about trauma more difficult. A federally funded high school education program in development, called First Aid for Severe Trauma™ (FAST™), will teach students to aid the severely injured. The program will be offered in instructor-led, web-based, and blended formats. We created a program to prepare high school teachers to become FAST instructors via “virtual” in-person (VIP) instruction. We used a webinar followed by VIP skills practice, using supplies shipped to participants’ homes. To our knowledge, no prior studies have evaluated this type of mass, widely distributed, VIP education.

**Methods:**

This study is a prospective, single-arm, educational cohort study. We enrolled a convenience sample of all high school teachers attending FAST sessions at the Health Occupations Students of America–Future Health Professionals International Leadership Conference. Half of the participants were randomized to complete the Stop the Bleed Education Assessment Tool (SBEAT) prior to the webinar, and the other completed it afterward; SBEAT is a validated tool to measure learning of bleeding competencies. We then performed 76 VIP video-training sessions from June–August 2020. The FAST instructors assessed each participant’s ability to apply a tourniquet and direct pressure individually, then provided interactive group skills training, and finally re-evaluated each participant’s performance post-training.

**Results:**

A total of 190 (96%) participants successfully applied a tourniquet after VIP training, compared to 136 (68%) prior to training (P < 0.001). Participants significantly improved their ability to apply direct pressure: 116 (56%) pre-assessment vs 204 (100%) post-assessment (P < 0.001). The mean score for the SBEAT increased significantly from pre-training to post-training: 2.09 with a standard deviation (SD) of 0.97 to 2.55 post-training with a SD of 0.72 (P < 0.001).

**Conclusion:**

This study suggests that a webinar combined with VIP training is effective for teaching tourniquet and direct-pressure application skills, as well as life-threatening bleeding knowledge. VIP education may be useful for creating resuscitative medicine instructors from distributed locations, and to reach learners who cannot attend classroom-based instruction.

## INTRODUCTION

Trauma is the leading cause of death for Americans between the ages of 1–44 years old.^1^ The 180 school shootings during the past decade, as well as recent evidence demonstrating the utility of early hemorrhage control in preventing deaths, has increased interest in training laypeople to treat injuries in school settings.^2^–^6^ The challenge of educating the public to treat trauma increased since the coronavirus disease 2019 (COVID-19) pandemic began, especially since the normal classroom settings in which teachers and students learn were altered significantly.

In 2018 the Department of Homeland Security awarded a grant to the Uniformed Services University of the Health Sciences’ (USU) National Center for Disaster Medicine and Public Health (NCDMPH) to create a nationwide, high school trauma-education program. This program, called First Aid for Severe Trauma™ (FAST™), is being developed by clinical and educational experts in collaboration with the American Red Cross.^7^ FAST emphasizes recent military medical lessons, especially point-of-injury hemorrhage control championed by the national Stop the Bleed campaign.^8^–^10^

The FAST curriculum, which launches in August 2021, is designed to foster lifesaving knowledge and skills to aid a severely injured person prior to the arrival of an ambulance. It includes lessons about scene safety, effective communication among rescuers and with 911 dispatchers, differentiating life-threatening from non-life-threatening bleeding, using direct pressure and tourniquets to stop bleeding, and positioning of the injured. The FAST program will be offered in instructor-led, web-based, and blended (combination of web and instructor-led elements) formats. A 2020 study demonstrated high school students’ ability to learn hemorrhage control knowledge and skills via these three educational modalities.^11^ In this study, the students demonstrated strong proficiency for learning didactic content via all modalities. They also learned to apply tourniquets via all modalities, although the blended and instructor-led modalities led to better performance compared to the online-only version, which did not include skills practice.^11^ A 2018 study showed that adults also have an ability to learn tourniquet application knowledge and skills via web-based instruction.^12^

Prior to the FAST program’s nationwide launch in 2021, NCDMPH had planned to facilitate train-the-trainer sessions in June 2020 during the Health Occupations Students of America–Future Health Professionals International Leadership Conference (HOSA ILC), a live symposium with approximately 2000 health science teacher attendees, to prepare a nationwide group of teachers to become Red Cross FAST instructors. However, since the COVID-19 pandemic forced the HOSA ILC to become a virtual conference, we designed an educational program and accompanying research protocol for high school teachers to learn FAST concepts and materials as part of their FAST instructor training via “virtual” in-person(VIP) instruction.The process consisted of a group webinar followed by VIP instruction consisting of small-group skills practice via video calls using supplies shipped to the teachers’ homes. These small groups re-emphasized material from the didactic session, and then used hands-on training to ensure skill competency. To our knowledge, no prior studies have evaluated this type of mass, widely distributed, VIP trauma or resuscitative medicine education.

## METHODS

### Study Design

This study is a prospective, single-arm, educational cohort study. The USU Institutional Review Board reviewed and approved it as an exempt educational study (protocol DBS 2020.116).

### Study Setting and Population

Study enrollment occurred from June–August 2020. The didactics sessions were held during the virtual HOSA ILC from June 23–June 26, 2020, and the VIP skills training occurred during a series of 76 small-group video conference sessions from June 30–August 7, 2020. High school health education teachers who self-selected for a “train-the-trainer” session at the HOSA ILC were eligible to become provisional FAST instructors.

### Study Protocol

The provisional FAST instructor training consisted of two components: a webinar for didactic material; and a VIP hands-on skill component. After attending both sessions, participants received a completion certificate. Following the final FAST course release in 2021, provisional instructors can become certified Red Cross FAST instructors after completing an online bridge training that discusses specific course policies using the finalized content.

High school teachers attending the HOSA ILC signed up for the FAST train-the-trainer sessions while registering for the conference online. Session attendees were not required to participate in the research, there was no cost to attend the sessions, and participants received no compensation. After registration, we emailed attendees who signed-up for train-the-trainer sessions to ask whether they would participate in the research. Inclusion criteria included being a teacher signed up to attend a FAST train-the-trainer session. After reviewing the study’s information sheet, 14 teachers declined to participate in the research, and all others assented to participate.

Participants provided demographic information and then received an appointment for one of five FAST webinars. At enrollment, half of the participants were randomly selected to complete the Stop the Bleed Education Assessment Tool (SBEAT) prior to each webinar, while the remaining half of the participants were instructed to complete the SBEAT within 48 hours after attending the webinar. We employed the SBEAT, which was previously validated in the general population, to measure learning outcomes for life-threatening bleeding competencies.^13^,^14^ We elected to randomize participants to complete either the pre-or post-test SBEAT, which is supported by SBEAT’s Rasch analysis, in an effort to avoid biasing results of the post-test.^15^ By not priming learners for the upcoming webinar with a pre-test, we sought to increase the external validity of our results. Furthermore, we predicted a higher completion rate if participants were asked to complete only a single SBEAT test, rather than two. We used block randomization to create an equal sample size in the pre- and post-test SBEAT groups. Each didactic session was divided into two blocks, which were thereafter randomized in an A-B-B-A order, with “A” representing pre-test and “B” representing post-test (ie, the first block of the first didactic session and the second block of the second didactic session were both assigned to take the pre-test SBEAT). An a priori power analysis was not performed, as we enrolled a convenience sample of all participants who enrolled in the FAST training.

The webinar included a standardized PowerPoint (Microsoft Corporation, Redmond, WA) lecture with embedded videos, animations, and images derived from the draft FAST materials, as well as an interactive question-and-answer session. The sessions lasted approximately two hours and were all taught by the same emergency physician who is a FAST curriculum expert. Following the completion of the webinar, participants were scheduled for the VIP skills training. Military medical students at USU who had been trained as FAST instructors previously, hosted VIP hands-on skills sessions with groups of three to five participants per session. One or two instructors led each session, and all sessions lasted about an hour. The sessions occurred in a standardized format, and instructors used scripts for consistency. Training supplies, including a windlass rod tourniquet (a generation 6 or 7 Combat Application Tourniquet) and a limb simulator were shipped to participants prior to the training. The study team emailed Google Meet or Zoom weblinks for the sessions to participants in advance of their sessions.

At the beginning of each VIP session, the FAST instructors assessed each participant’s ability to apply a tourniquet and direct pressure correctly by meeting with each participant in separate virtual rooms. The participant applied the tourniquet to the limb simulator. The instructor, watching via webcam, determined whether the tourniquet application was successful by using a checklist to assess the technique, positioning, and tightness. A similar checklist has been used in multiple prior studies to evaluate proper tourniquet application.^11^,^12^,^16^,^17^ The instructor did not provide feedback or corrections during this pre-assessment phase. Next, the instructor evaluated each participant’s ability to perform direct pressure by observing the participant apply direct pressure to the limb simulator using the “cardiopulmonary resuscitative (CPR) posture” taught during the webinar.^18^ An instructor scored the participant’s direct pressure as successful, if he or she applied pressure to the wound using the appropriate posture, and the limb simulator was deformed from body weight.

After the pre-assessment, instructors facilitated an interactive group session with the participants to describe and demonstrate the skills of tourniquet and direct pressure application, highlight points from the didactic webinar, and allow for group practice and clarifying questions with instructor feedback. The primary focus of these VIP sessions was learner skill acquisition. Following the group session, each participant returned to a separate virtual room with a single instructor for repeat evaluation of the participant’s tourniquet and direct pressure application skills. The instructor performed the post-assessment using an identical checklist to the pre-assessment. Each participant had up to two opportunities to perform tourniquet and direct pressure application correctly. If a participant failed after the first attempt, the instructor provided corrective instruction prior to performing the second attempt. Participants who did not perform a skill successfully after two attempts were remediated to ensure they could perform the skills; however, the performance was counted as a failure for the purposes of study enrollment.

### Key Outcome Measures

The primary outcome of the study was successful performance of tourniquet and direct pressure application. Secondary outcomes included performance on the SBEAT, time for tourniquet application, and reasons for tourniquet application failure.

### Data Analysis

The data gathered about tourniquet application, specifically the location, the tightness, and the completion of all steps, and data about direct pressure, specifically regarding correct CPR posture, are presented as binary outcomes, with attempts being either successful or not successful. The amount of time participants took to apply a tourniquet is presented as a continuous outcome. Comparisons between pre- and post-training skill difference were conducted using a paired-sample *t*-test for the continuous outcome and chi-square tests for the binary outcomes. We conducted all analyses using two-tailed tests, and *P* values less than 0.05 were considered statistically significant. Analysis was performed using IBM Statistical Package for the Social Sciences (SPSS) software version 25.0 (IBM Corporation, Armonk, NY).

Demographic information is presented as counts and percentages for categorical data and as means and standard deviations (SD) for continuous data. We transformed SBEAT item-response data, which are non-equal scores, to a linear measure through Rasch modeling, yielding individual person measures using Winsteps 2020 software (Zoominfo Technologies, LLC, Beaverton, OR). Rasch modeling calculates linear person ability estimates (interval scale) that can then be statistically assessed. Scores can range from −4 to 4, where 0 refers to a 50/50 probability of success.^19^ Independent *t-*tests were then performed in IBM SPSS software version 26.0 to identify differences between groups in pre- and post-test scores.

## RESULTS

**Figure 1:**
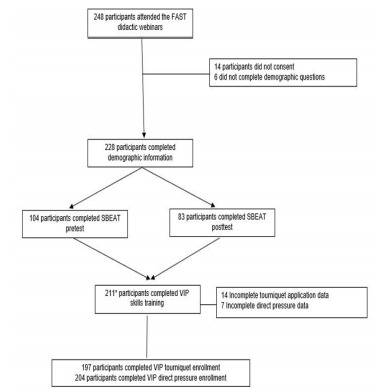
Study flow. *FAST*, First Aid for Severe Trauma; *SBEAT*, Stop the Bleed Education Assessment Tool; *VIP*, virtual-in-person.

**Figure 2:**
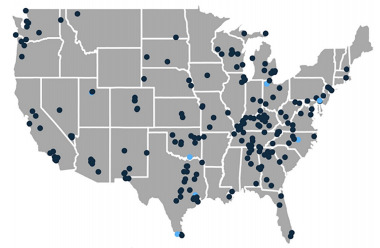
Locations of high school teachers trained during study. *Light color dots indicate multiple people in the same area.

A total of 248 high school teacher participants attended the FAST webinars. Of these, 228 participants completed the demographic information questions, 211 completed the VIP skills training, and 187 completed the SBEAT (Figure 1). Of the participants 208 (91%) were female, and the average age was 46 with a range of 23–70 years old (Table 1). The participants had an average of 12 years of teaching experience with a SD of eight years, and 83% had bachelor’s or master’s degrees. Teachers from 45 of the United States and Washington, DC, participated in the study (Figure 2).

A total of 190 (96%) participants successfully completed tourniquet application after VIP training, compared to 136 (68%) prior to training (*P* < 0.001) (Table 2). Participants also significantly improved their ability to apply direct pressure correctly with 116 (56%) participants performing it correctly during the pre-assessment and 204 (100%) performing direct pressure correctly after VIP training (*P* < 0.001). The primary reason participants did not apply the tourniquet correctly was due to inadequate tightness. This error decreased significantly post-training (3 [2%]) compared to pre-training (36 [18%]) (*P* < 0.001). The mean time to apply a tourniquet decreased from 42 seconds pre-training to 29 seconds post-training (*P* < 0.001).

Of the 187 participants who completed the SBEAT, 104 completed the pre-test and 83 completed the post-test. The mean score for SBEAT at pre-training was 2.09 with a SD of 0.97 and the mean score for SBEAT at post-training was 2.55 with a SD of 0.72, which was a statistically significant difference (*P* < 0.001).

## DISCUSSION

This study suggests that synchronous web-based and “virtual” hands-on training are effective for teaching the technical skills of tourniquet and direct pressure application, as well as the cognitive knowledge of life-threatening bleeding, contained in the FAST course. Sixty-eight percent of study participants applied tourniquets correctly following the webinar didactic, and prior to the VIP training. This is similar to the 75% of successful tourniquet applications found in a 2018 study assessing the public’s ability to apply tourniquets after web-only training.^12^ Following the VIP skills training, the participants demonstrated statistically significant improvement of their tourniquet skill demonstrations with 96% of participants applying a tourniquet correctly, and 100% of participants performing direct pressure application correctly. This is similar to the 88% of successful tourniquet applications found after in-person training following a Stop the Bleed course.^20^ Unfortunately, due to the COVID-19 pandemic, we could not execute the desired in-person training control arm of this study, and its absence prevents us from making conclusions about non-inferiority or superiority of the VIP training to in-person training.

The SBEAT analysis showed statistically significant improvement in participant knowledge demonstration from the pre- to post-test. The instrument noted good item separation from novice to expert in the knowledge and behaviors of life-threatening bleeding, which is the primary focus of the FAST course. Similar to previous studies assessing the public’s ability to learn Stop the Bleed knowledge with brief education, the significant difference between the pre- and post-test SBEAT scores in this study demonstrates that the public can learn hemorrhage control knowledge rapidly using a variety of modalities.^11^,^12^

This study provides the field of first aid and resuscitative education proof of concept that harmonization of knowledge and skills can be achieved through synchronous online modalities. The combination and sequence of the introduction of FAST concepts via webinar followed by individual practice and validation via VIP skills practice led to the improvement of learning outcomes. Training and educational organizations that previously relied on face-to-face interactions can use this approach to maintain or expand their cohort of educators. Specific to the development of FAST instructors, this process may serve as a multiplier to disseminate training more broadly by reducing costs and increasing access, which is a known barrier to implementing resuscitative medicine programs.^21^ Furthermore, VIP training can reduce the need for classroom-based training and its associated challenges, such as finding childcare and transportation, even during non-pandemic times.^22^ The VIP concept could be tailored to include additional elements. As an example, since our study participants were all active high school teachers, our VIP train-the-trainer process did not include a requirement for participants to “teach” the new material they had just learned. A “demonstration of teaching” could be adopted readily to a VIP model if needed.

This proof of concept of VIP skills training may have significant utility, especially during this time of a global pandemic without a clear endpoint in sight. There is an ongoing need for the public to complete a variety of types of resuscitative medicine and public health training, such as CPR and workplace first aid, in addition to hemorrhage control. This study could serve as a model for other trainings to be conducted from the safety and convenience of people’s homes. The study is consistent with a growing body of literature that alternative modalities of education, such as airport kiosks, videos, and just-in-time education, are feasible for teaching resuscitative medicine knowledge and skills.^11^,^12^,^16^,^17^,^23^–^25^

## LIMITATIONS

This study has several limitations. The low-fidelity limb simulator used in the study could not provide real-time pressure data for direct pressure application, although we think it supported adequate remote skill assessment. While the study design increased external validity by reducing test-retest bias and participant priming for the course, having two separate groups take the pre- and post-test limited the ability to conclude that the groups taking the SBEAT were not significantly different. The skill teaching and assessment were not blinded to the participant or evaluator, which may have introduced bias. Since the pandemic precluded the ability to implement an in-person education control arm, no direct comparison to another educational modality could be made. However, given the extremely high performance of participants in this study, as well as supporting data from other studies, we have little reason to suspect that in-person training would be dramatically different.^11^,^20^

This study did not assess knowledge or skill retention. Multiple studies have demonstrated the rapid degradation of resuscitative medicine skills by the public, including an in-person hemorrhage control education study that showed a decrease in successful tourniquet application of about 40% in just a few months.^20^,^25^ It would be important to consider the need for re-training, periodic assessment, or learning adjuncts for anyone trained via a VIP instructional modality. The Red Cross FAST course will require instructors to teach a minimum number of courses and re-certify periodically to maintain credentials.

Generalizability across the US population may also be limited as the participants were predominantly female, White, well educated, had access to remote learning technology, and worked as health science educators. The study also trained people who desired to become instructors, rather than an undifferentiated learner population, so the results may be biased by a highly motivated learner population. While these results may or may not be directly translatable to other populations, it is likely that other groups of learners desiring to become instructors would be similarly motivated. It is also noted that studies of the undifferentiated general public have demonstrated similar performance in knowledge and skills after web and in-person training.^12^,^20^

## CONCLUSION

This study suggests that a didactic webinar combined with “virtual” in-person skills training is effective for teaching the cognitive hemorrhage-control knowledge and the technical skills of tourniquet and direct pressure application. This VIP educational modality may be particularly useful for building or expanding new resuscitative medicine instructors from broad geographic locations, as well as for educators who would like to reach a broader audience than those who can or will attend classroom-based instruction.

**Population Health Research Capsule**

What do we already know about this issue?

*Prompt hemorrhage control with a limb tourniquet can be lifesaving. Previous studies have shown lay adults’ ability to learn tourniquet application via in-person training*.

What was the research question?

*Can laypeople learn bleeding control knowledge and skills from instructor-led virtual instruction?*

What was the major finding of the study?

*A webinar combined with “virtual” in-person skills training is effective for teaching hemorrhage control skills*.

How does this improve population health?

*Instructor-led virtual education may reach more learners than classroom-based instruction alone, thereby enhancing efforts to teach lifesaving medical skills*.

## Figures and Tables

**Table 1:** Participant demographics.

	Mean (SD) or n (%)
Age	46 (10)
Years of teaching experience	12 (8)
Gender	
Male	19 (8)
Female	208 (91)
Race	
White	207 (91)
Black or African American	9 (4)
Asian	7 (3)
American Indian or Alaska Native	1 (0)
Native Hawaiian and Pacific Islander	1 (0)
Other or multiple race	3 (1)
Ethnicity	
Hispanic origin	15 (7)
Not hispanic origin	209 (92)
Highest level of education	
High school	6 (3)
Bachelor’s degree	81 (36)
Master’s degree	110 (48)
Doctorate degree	13 (6)

*SD*, standard deviation.

**Table 2:** Results for primary and secondary outcomes.

Binary variables	Pre-training n (%)	Post-training n (%)	P-value
Successful tourniquet application	136 (68%)	190 (96%)	< .001
Reasons for failed tourniquet application			
Incorrect location	24 (12%)	4 (2.0%)	< .001
Inadequate tightness	36 (18%)	3 (2%)	< .001
Failure to complete all steps	36 (18%)	3 (2%)	< .001
Correct direct pressure application	116 (56%)	204 (100%)	< .001

Continuous variables	Mean (SD)	Mean (SD)	P-value

SBEAT score	2.09 (0.97)	2.55 (0.72)	< .001
Time of tourniquet application (seconds)	41.55 (25.03)	28.60 (12.66)	< .001

*SBEAT*, Stop the Bleed Education Assessment Tool.
